# Profound Impact of Hfq on Nutrient Acquisition, Metabolism and Motility in the Plant Pathogen *Agrobacterium tumefaciens*

**DOI:** 10.1371/journal.pone.0110427

**Published:** 2014-10-17

**Authors:** Philip Möller, Aaron Overlöper, Konrad U. Förstner, Tuan-Nan Wen, Cynthia M. Sharma, Erh-Min Lai, Franz Narberhaus

**Affiliations:** 1 Microbial Biology, Ruhr University Bochum, Bochum, Germany; 2 Research Center for Infectious Diseases (ZINF), Julius-Maximilian's University of Würzburg, Würzburg, Germany; 3 Institute of Plant and Microbial Biology, Academia Sinica, Taipei, Taiwan; Univ. of Edinburgh, United Kingdom

## Abstract

As matchmaker between mRNA and sRNA interactions, the RNA chaperone Hfq plays a key role in riboregulation of many bacteria. Often, the global influence of Hfq on the transcriptome is reflected by substantially altered proteomes and pleiotropic phenotypes in *hfq* mutants. Using quantitative proteomics and co-immunoprecipitation combined with RNA-sequencing (RIP-seq) of Hfq-bound RNAs, we demonstrate the pervasive role of Hfq in nutrient acquisition, metabolism and motility of the plant pathogen *Agrobacterium tumefaciens*. 136 of 2544 proteins identified by iTRAQ (isobaric tags for relative and absolute quantitation) were affected in the absence of Hfq. Most of them were associated with ABC transporters, general metabolism and motility. RIP-seq of chromosomally encoded Hfq^3xFlag^ revealed 1697 mRNAs and 209 non-coding RNAs (ncRNAs) associated with Hfq. 56 ncRNAs were previously undescribed. Interestingly, 55% of the Hfq-bound ncRNAs were encoded antisense (as) to a protein-coding sequence suggesting that *A. tumefaciens* Hfq plays an important role in asRNA-target interactions. The exclusive enrichment of 296 mRNAs and 31 ncRNAs under virulence conditions further indicates a role for post-transcriptional regulation in *A. tumefaciens*-mediated plant infection. On the basis of the iTRAQ and RIP-seq data, we assembled a comprehensive model of the Hfq core regulon in *A. tumefaciens*.

## Introduction

Post-transcriptional gene regulation is a key strategy in the dynamic adaption to changing environmental conditions. In bacteria, small regulatory RNAs (sRNAs, non-coding RNAs, ncRNAs) rapidly adjust gene expression to the physiological needs and have also been implicated in virulence control [1], [2]. Bacterial ncRNAs usually influence translation or stability of their cognate mRNA target. Translational regulation occurs via blockage or release of the Shine-Dalgarno (SD) sequence on the mRNA [3], [4]. Further, the double-stranded ncRNA-mRNA duplex is often recognized and degraded by RNase III [5], [6], [7], [8]. Rapid and dynamic adjustment of the cellular RNA pool also involves other RNases (e.g. RNaseE, PNPase) that interact with single-stranded RNAs and modulate processing, degradation and general quality control of ncRNAs and mRNAs.

ncRNAs are distinguished according to their location on the genome in anti-sense (asRNA) or trans encoded small RNAs (sRNAs). asRNAs partly or completely overlap with their target mRNAs encoded on the complementary strand. Due to the perfect sequence complementarity, asRNA-mRNA interactions are believed to be rather Hfq-independent [9], [10], [11], [12], [13]. In contrast, sRNAs are encoded in intergenic regions and usually share imperfect sequence complementarity with their target mRNA. The association of sRNAs with their target mRNAs is often promoted by the RNA-chaperone Hfq [14].

Hfq is a homohexameric (L)Sm-like protein and was first identified as a host factor for phage Qβ in *Escherichia coli* [15]. The ring-shaped protein exposes a proximal, a distal and a lateral RNA-binding surface, allowing specific binding of RNA molecules [16], [17]. ncRNAs bind primarily to the proximal face via U-rich internal regions and 3′ poly-U tails [18], while 5′ A-rich mRNA sequences, including ARN-motifs (A *adenosine*, R *purine*, N *any nucleotide*), bind to the distal surface [4], [19]. The lateral surface supports sRNA-binding in *E. coli* [20]. Aside from mediating association of sRNA and mRNA, Hfq also directly influences its interaction partners [21]. On the one hand, Hfq-binding prohibits RNase-dependent decay, while on the other hand, ongoing interaction with Hfq promotes polyadenylation and subsequent RNA decay [22], [23], [24]. Deletion of *hfq* is usually accompanied by pleiotropic phenotypes and often results in reduced growth, motility and stress tolerance, as shown for numerous bacteria including the α-proteobacteria *Brucella abortus* [*25*] *, Rhodobacter sphaeroides* [11], *Sinorhizobium meliloti* [26], [27], [28], [29], *Rhizobium leguminosarum* [30] and *Agrobacterium tumefaciens* [31]. Most strikingly, Hfq is required for successful host-microbe interactions in several symbiotic and pathogenic bacteria [32].

Deletion of *hfq* in the phytopathogen *A. tumefaciens* leads to reduced viability and a severe reduction in plant infection efficiency but only eight direct targets are known so far [31]. The genome of *A. tumefaciens* consists of a circular chromosome, a linear chromosome, the At-plasmid and the Ti (*tumor-inducing*)-plasmid [33]. *A. tumefaciens* is capable of interkingdom DNA transfer, leading to tumor formation on infected plants [34]. Virulence is induced by exposure of the bacteria to plant wound molecules (e.g. acetosyringone, low pH, monosaccharides). Signal perception leads to the activation of virulence (*vir*) gene expression and transport of a single-stranded DNA fragment (T-DNA) into the plant cell via the VirB/D4 type-4 secretion system (T4SS). Integration of the T-DNA into the plant chromosome results in phytohormone and opine biosynthesis. Two sequencing studies of the *A. tumefaciens* RNome identified 621 transcripts not dedicated to protein-coding thus constituting a large pool of potential regulatory RNAs [35], [36]. These ncRNAs are distributed among all four replicons. Thirty-six (6%) of the identified ncRNAs were verified by Northern blot experiments, including the ABC-transporter regulator AbcR1, which turned out to be Hfq-dependent [35], [36], [37], [38]. AbcR1 participates in bacteria-plant interactions by influencing susceptibility of *A. tumefaciens* to the plant defense molecule γ-aminobutyric acid (GABA) via negative regulation of *atu2422* encoding the periplasmic binding protein of the GABA uptake system. At least 15 additional AbcR1 targets suggest an extensive sRNA regulon [38]. Apart from that, it is still unclear how many additional ncRNAs and mRNAs are Hfq-dependent in *A. tumefaciens.* Despite the rapidly growing number of ncRNAs, identification of the corresponding mRNA target is still challenging. Determination of Hfq-bound mRNAs and ncRNAs has become an applicable technique to narrow down the involved transcripts and increase accuracy of subsequent analysis, e.g. biocomputational predictions.

To determine the underlying molecular mechanism of Hfq-dependent regulation in *A. tumefaciens* and to globally identify Hfq-dependent transcripts, we analyzed the Δ*hfq* global proteome by iTRAQ and the Hfq-bound RNA-interactome by RIP-seq. Our results demonstrate a major impact of Hfq on regulatory networks balancing nutrient acquisition, cellular metabolism and motility. Further, we revealed extensive binding of asRNAs to Hfq and validated the influence on asRNA-mediated regulation. Hfq also bound mRNAs of the major virulence genes thus indicating a distinct role in plant infection.

## Materials and Methods

### Bacterial strains, plasmids, media

All strains and plasmids used in this study are listed in Table S1. *Agrobacterium tumefaciens* C58 strains were cultivated at 30°C in YEB complex medium or AB minimal medium (pH 5.5, supplemented with 1% (w/v) glucose) [39]. For virulence induction overnight cultures of *A. tumefaciens* were inoculated in AB medium to an OD_600 nm_ of 0.1 and grown for 6 h at 30°C. Virulence gene expression was induced by addition of 0.1 mM acetosyringone (Sigma-Aldrich, Germany) and further cultivation at 23°C for 16 h. Non virulence induced cultures were treated with equal volumes of DMSO. For cultivation during mutagenesis, *A. tumefaciens* cells were grown in Luria-Bertani (LB) medium [40], supplemented with either 10% (w/v) sucrose or 50 µg ml^−1^ kanamycin (Km).

### Chromosomal integration of *hfq* ^3xFlag^

The *hfq* (*atu1450*) gene of the *A. tumefaciens* C58 circular chromosome was tagged with a *3xFlag* at the 3′ end. For mutagenesis plasmid construction, a region upstream of *hfq* including its open reading frame without the TGA stop codon was amplified by PCR using primers *hfq*_up_*Pst*I_fw and *hfq*_up_*Sal*I_rv (Table S2). The 3xFlag tag was amplified from *E. coli* MG1655 *kdtA*::3XFLAG chromosomal DNA [41] with primers 3xFlag_*Sal*I_fw and 3xFlag_*Acc*65I inserting a TGA stop codon at the 3′ of the 3xFlag sequence. The *hfq* downstream region was amplified using primers *hfq*_down_*Acc*65I_fw and *hfq*_down_*EcoR*I_rv. The resulting PCR fragments *Pst*I_*hfq*_up_*Sal*I, *Sal*I_3xFlag_*Acc*65I and *Acc*65I_*hfq*_down_*EcoR*I were subsequently ligated into pK19*mobsacB* suicide vector [42], resulting in *hfq*_up_3xFlag_down mutagenesis plasmid. The plasmid was transformed into *A. tumefaciens* C58 wild-type cells by electroporation (800 Ω, 25 µF, 2 kV) and selected for homologous recombination by Km resistance on LB+Km agar plates. Single colonies were grown overnight in LB medium without antibiotics and plated on LB agar plates containing sucrose. Double cross over events resulted in sucrose tolerant and Km sensitive colonies. Putative mutants encoding *hfq* ^3xFlag^ on the chromosome were verified by Southern blot analysis [40].

### Total RNA preparation and Northern-blot analysis

RNA was isolated from *A. tumefaciens* strains as described in [31] by the hot acid phenol method [43]. Northern blot analyses were performed as previously described [37]. For RNA detection 8 µg (for mRNAs) to 10 µg (for sRNAs) total RNA were separated on agarose or polyacrylamide gels respectively, blotted on Hybond-N membranes (GE Healthcare, USA) and hybridized with specific DIG-labelled (Roche Applied Sciences, Germany) RNA probes. Oligonucleotides used for RNA probe synthesis are listed in Table S2. For signal detection a Hyperfilm ECL (GE Healthcare, USA) system was used.

### Hfq^3xFlag^ co-immunoprecipitation

Co-immunoprecipitation (coIP) experiments of Hfq^3xFlag^ and bound RNAs were based on the procedure described in [9], [44] with minor changes. 100 ml of *A. tumefaciens* wild-type and *hfq* ^3xFlag^ cultures grown to OD_600 nm_ 0.5 and 1.0 in YEB medium or under non-induced (+DMSO) and virulence-induced (+acetosyringone) conditions in AB medium were harvested and resuspended in 2 ml ice-cold lysis buffer (20 mM Tris (pH 7.5), 150 mM KCl, 1 mM MgCl_2_, 1 mM DTT). Cells were disrupted by French Press (3 passes, 16,000 psi) and centrifuged at 10,000×g, 4°C, for 1 h. 10 ng monoclonal ANTI-3XFLAG M2 antibody (Sigma-Aldrich, Germany) were coupled to 50 µl Dynabeads Protein G (ThermoFisher Scientific, Life Technologies, USA) as described in the instruction manual, and incubated with the supernatant (3 h, 4°C). Dynabeads were separated on a magnet and washed 5x with PBS buffer. RNA was isolated using phenol/chloroform/isoamylalcohol and chloroform/isoamylalcohol followed by precipitation with ethanol and sodium acetate. After precipitation remaining DNA was digested by DNaseI (ThermoFisher Scientific, Life Technologies, USA) and RNA was precipitated as described before.

### RNA-sequencing

Preparation of cDNA libraries was performed at Vertis Biotechnology AG (Germany). Equal amounts of RNA samples were poly(A)-tailed and 5′-PPP were removed. The RNA adapter was ligated to the RNA 5′-monophosphate and reverse transcription was performed with oligo(dT)-adapter primers resulting in first-strand cDNA. Higher yields of DNA (20–30 ng µl^−1^) were gained by further PCR-based amplification using primers designed for TruSeq according to recommendations for Illumina (HiSeq). For multiplex sequencing a library specific barcode was part of the 3′- sequencing adapter. Purification was achieved using the Agencourt AMPure XP kit (Beckman Coulter Genomics, USA), followed by capillary electrophoresis. Final cDNAs were sequenced using a HiSeq 2500 machine in single-read mode and running 100 cycles. Raw (de-multiplexed) reads and normalized coverage files were deposited in the Gene Expression Omnibus (GEO) of the National Center for Biotechnology Information [45] and are accessible via the GEO accession GSE59123 (http://www.ncbi.nlm.nih.gov/geo/query/acc.cgi?acc=GSE59123).

### RNA-sequencing data analysis

Illumina reads in FASTQ format were trimmed (cut-off phred score 20) by the fastq_quality_trimmer program from FASTX toolkit 0.0.13 (http://hannonlab.cshl.edu/fastx_toolkit/). Further processing was performed using “create”, “align” and “coverage” subcommands of the READemption tool 0.2.6 with default parameters (Förstner et al., submitted). Poly(A)-tail sequences were removed and sequences shorter than 12 nt were eliminated. Collections of the remaining reads were mapped to the *A. tumefaciens* C58 reference genome (NC_003062.2, C_003063.2, NC_003064.2, NC_003065.3 - downloaded from the NCBI ftp server) using segemehl [46]. Mapping statistics (input, aligned, uniquely aligned reads etc.) are listed in Table S3. The numbers of aligned reads per nucleotide were represented by coverage plots (wiggle format) and visualized in the Integrated Genome Browser [47]. Normalization was performed based on the total number of reads aligned from the respective library. Multiplication of the corresponding graphs by the minimum number of mapped reads calculated from all libraries prevented rounding of small numbers to zero. For read quantification, annotation files in GFF3 format (accession numbers mentioned above) were obtained from the NCBI ftp server. Intergenic and antisense regions fulfilling the Hfq-dependency criteria (see below), were manually curated and regions were adjusted according to IGB browser information from all libraries. The number of reads overlapping with annotation entries was calculated using the READemption “gene_quanti” subcommand. Reads overlapping in sense, anti-sense of all annotations were considered and counted separately.

### Hfq dependent RNAs

5459 genes, 621 ncRNAs and 819 transcriptional start sites (TSS) identified in previous studies were included in the annotation [35], [36]. Protein-coding sequences with so far undefined TSS were extended by virtual 54 nt at the 5′ UTRs, as described by [48]. All transcripts were additionally extended by virtual 20 nt at the 3′ UTR, applying the minimal transcriptional unit model (Fig. S1). Hfq enriched RNAs were further subjected to manual curation and reads overlapping the defined 5′ or 3′ features were merged with the cognate transcript to gain more accurate transcriptional units. RNAs with a minimal raw read count (RRC) of 50 and at least 2-fold enrichment (after a normalization by the total number of aligned reads of each library) in the Hfq^3xFlag^ library compared to the corresponding Hfq^WT^ library were considered enriched.

### Identification of new ncRNAs

Transcripts fulfilling the Hfq-dependency criteria but not dedicated to any annotated feature were classified ncRNAs, when they reached a minimal length of 50 nt and a total number of aligned reads of 50 [48]. Transcripts not overlapping any feature were classified as trans sRNAs, while transcripts partly or completely overlapping a feature in anti-sense orientation were classified as anti-sense RNAs. Newly identified sRNAs were named (*Agrobacterium tumefaciens* Hfq associated ncRNA) AhaR_X_Y, with “X” varying for the *A. tumefaciens* genomic replicons (C: *circular chromosome*, L: *linear chromosome*, At: *At-plasmid*, Ti: *Ti-plasmid*) and “Y” for ongoing numbering.

### Bacterial growth, protein isolation and trypsin digestion for iTRAQ

*A. tumefaciens* C58 WT and Δ*hfq* strains were grown to OD_600 nm_ 1.5 in YEB medium (30°C). Cells were harvested (4,000×g, 20 min, 4°C) and washed 2x in Tris-Cl (50 mM Tris-HCl, pH 7.5, 200 mM KCl) and suspended in Lysis buffer (50 mM Tris-HCl, pH 7.5, 200 mM KCl, 1 mM PMSF, 1x Protease inhibitor mix) to a final OD_600 nm_ of 10. Cells were disrupted by French Press (3 passes, 16,000 psi) and lysates were centrifuged 2x (10,000×g, 10 min, 4°C). Supernatants were precipitated overnight with 6 volumes pre-chilled TCA (10 w/v)/acetone. Precipitated proteins (10,000×g, 25 min, 4°C) were washed 3x with 85% cold acetone, dried and resuspended in urea buffer (8 M urea, 50 mM Tris-Cl, pH 8.5). Protein concentrations were measured using Pierce 660 nm protein Assay kit (Thermo Scientific, Germany). Protein isolation, trypsin digestion and subsequent peptide treatment were performed as previously described in [49] with minor modifications. Total proteins (100 µg) were reduced by addition of DTT to a final concentration of 10 mM (1 h, 37°C). Further treatment with a final concentration of 50 mM iodoacetamide (30 min, RT, dark) was followed by consumption of any free iodoacetamide by 10 mM DTT (1 h, RT, dark). Proteins were diluted with 50 mM Tris-Cl_,_ pH 8.5 (final urea concentration less than 4 M) and digested with 250 units/ml Benzonase (Sigma-Aldrich, Germany) (RT, 2 h), followed by Lys-C (Wako, Japan) digestion (1∶200 (w/w), 4 h, RT). Proteins were diluted with 50 mM Tris-Cl pH 8.0 (final urea concentration less than 1 M) and digested with 2 µg of modified trypsin (Promega) (1∶50 (w/w), 37°C, overnight). The peptide solution was acidified with 10% trifluoroacetic acid, desalted on an Oasis HLB cartridge (Waters, USA) and dried by SpeedVac.

### Labeling of peptides with iTRAQ reagents and fractionation

Peptide pellets were dissolved in iTRAQ dissolution buffer and labeled with iTRAQ reagents according to the manufacturer’s manual (Applied Biosystems). Wild-type samples were labeled with reagent 114 while Δ*hfq*-mutant proteins were labeled with reagent 115 (1 h, room temperature). iTRAQ labeled peptides were combined and further fractionated using a strong cation-exchange column (SCX, PolySulfoethyl A, 4.6×100 mm, 5 µm, 200 Å, PolyLC Inc.) on HPLC. The SCX chromatography was performed with an initial equilibrium buffer A (10 mM KH_2_PO_4_, 25% ACN pH 2.65), a 40 min linear gradient from 0% to 50% buffer B (1 M KCl in buffer B, pH 2.65), 5 min in 50% buffer B, 1 min in a linear gradient from 50% to 100% buffer B at a 1 ml min^−1^ flow rate. According to the peak area (Abs 214 nm) the collected fractions were pooled into five final fractions. Samples were desalted using an Oasis HLB cartridge (Waters, USA) prior to LC-MS/MS.

### LC-MS/MS analysis

SCX fraction samples were resuspended (0.1% formic acid) and analyzed using a nanoUPLC system (nanoAcquity, Waters, USA) coupled to an LTQ Orbitrap Elite hybrid mass spectrometer (Thermo Scientific, Germany). A C18 capillary column (1.7 µM particle size, 75 µM×250 mm, BEH130, Waters, USA) was used to separate peptides with a 120 min linear gradient from 5% to 40% ACN at a flow rate of 300 ml min^−1^. The LTQ Orbitrap Elite MS was operated in the data-dependent mode with top 15 ions (charge states ≥2) from the MS survey scan selected for subsequent HCD activation and MS/MS acquisitions in the Orbitrap cell. For MS and MS/MS the FT Orbitrap m/z range was set to 350–1600 with a resolving power of 120,000 and AGC of 500,000. HCD was set to MSn AGC target = 50,000 with a minimal signal of 5,000. Isolation width was 1.2 with an NCE of 35%, activation time of 0.1 ms and a resolving power of 15,000. Data dependent settings for dynamic exclusion were 1 for repeat count, 15 sec repeat duration and 90 sec for exclusion duration. Peptide identification was performed using the Proteome Discoverer software (v1.3, Thermo Fisher Scientific, USA) with SEQUEST and Mascot (v2.3, Matrix Sciences) search engines. MS data were searched against the *Agrobacterium tumefaciens* C58 protein sequence database (http://www.ncbi.nlm.nih.gov/). Peptides with 2 maximum missed cleavage sites after trypsin digestion were analyzed with a precursor mass tolerance of 10 ppm and a fragment mass tolerance of 50 mmu. Dynamic modifications were oxidation (M) while static modifications included carbamidomethyl (C) and 4plex-iTRAQ tags (N-terminus and K). The identified peptides were validated using Percolator algorithm which automatically conducted a decoy database search and rescored peptide spectrum matches (PSM) using q-values and posterior error probabilities. All PSMs were filtered with a q-value threshold of 0.01% (1% false discovery rate) and proteins were filtered with a minimum of 2 distinct peptides identified per protein. For iTRAQ quantification, the ratios of iTRAQ reporter ion intensities in MS/MS spectra (m/z: 114, 115) from raw datasets were used to calculate the fold changes between control and treatment. Only unique peptides were used for peptide/protein quantification. All peptide ratios were normalized by the median protein ratio. The protein ratio was calculated from three biological replicates.

### iTRAQ data analysis

Proteins with significantly changed abundance in the *hfq* deletion strain were selected as previously described in [50] with minor modifications. Mean and standard deviation (*SD*) from ln ratios of all 2544 identified proteins were calculated. A confidence interval of 95% (Z score = 1.96) was used to select proteins with a distribution outside the main distribution. For down-regulated proteins the confidence interval was 0.016 (*mean ratio*)–1.96×0.413 (*SD*), corresponding to a protein ratio of 0.4524. Proteins up-regulated were similarly calculated (*mean ratio* +1.96×*SD*), corresponding to a protein ratio of 2.2823. Therefore, the cut-off value for down-regulated proteins was set 0.45-fold and for up-regulated proteins 2.3-fold. Proteins were considered significantly regulated when reaching the cut-off value and a variability of less than 40% between the replicates. For protein ratios that failed the variance criterion, a combined ratio was calculated (*combined ratio* = *ratio* +/− *ratio*×*variance*). For up-regulated proteins variance was subtracted from the ratio, for down-regulated proteins variance was added to the ratio. Combined ratios that reached the cut-off criterion were also considered statistically significant. By this, proteins with higher variances were included when they explicitly reached the cut-off criteria for up- or down-regulation.

### Protein analyses by SDS PAGE and Western blot

Protein samples were separated by SDS gel electrophoresis on 12.5% polyacrylamide gels. Proteins were transferred to nitrocellulose membranes (Hybond-C, GE Healthcare, USA) by Western transfer using standard protocols [40]. 3xFlag protein fusions were detected using monoclonal ANIT-3XFLAG M2 antibody (Sigma-Aldrich) and the corresponding secondary goat anti-mouse IgG (H+L)-HRP conjugate (Bio-Rad, USA). Protein signals were visualized using Luminata Forte Western HRP substrate (Millipore). For signal detection the ChemiImager Ready system (Alpha Innotec) was used.

### Network prediction, statistics and visualization

Operons were predicted using (http://meta.microbesonline.org/operons/gnc176299.html) [51]. Proteins were clustered based on KEGG Brite ontology for *A. tumefaciens* C58 (http://www.kegg.jp/kegg/tool/map_brite2.html) [52], [53]. Statistical relevance of protein clustering was calculated using a two-tailed Fischer test [54]. Predictions of physical and functional protein interactions were performed using the String 9.1 webserver (http://string-db.org/) [55]. Results were visualized by Cytoscape 3.1.0 [56], [57]. Additional ncRNA nodes and corresponding edges were added manually.

## Results

### Differential abundance of Hfq-dependent proteins

**Figure 1:**
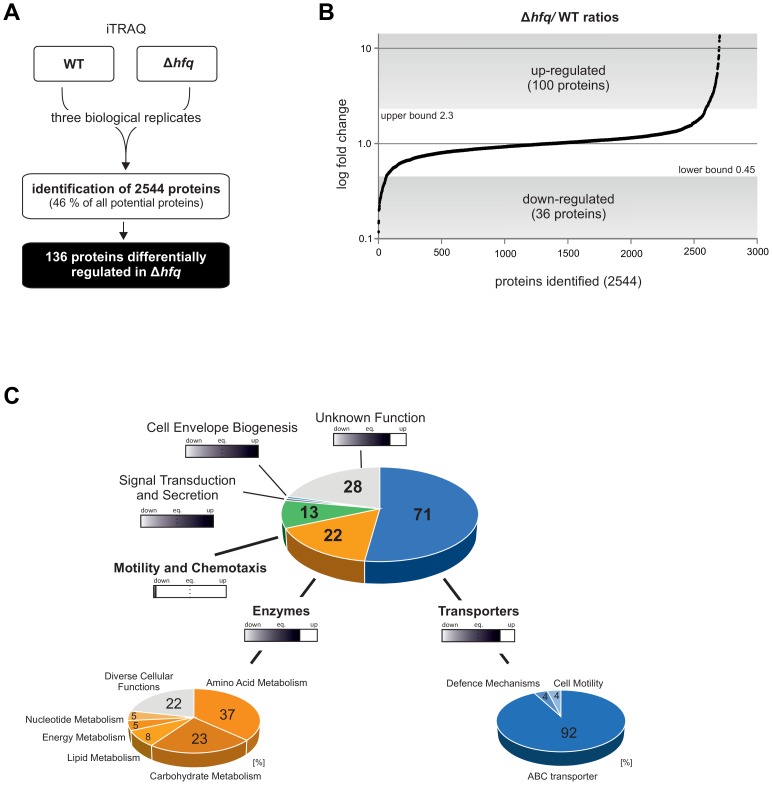
Quantitative proteomics of *A. tumefaciens* WT and Δ*hfq* mutant. **A)** iTRAQ experiments of 3 biological replicates from stationary phase cultures of WT and Δ*hfq* mutant revealed 136 proteins differentially expressed (2544 proteins identified). **B)** Distribution of all 2544 Δ*hfq*/WT logarithmic (log) fold-changes (FC). Calculating a confidence interval of 95% resulted in an upper bound of 2.3 and a lower bound of 0.45. 100 proteins were up-regulated (FC>2.3) and 38 down-regulated (FC<0.45) in absence of *hfq*. **C)** Classification of proteins into physiological relevant groups by KEGG ontology. Filled bars indicate up- or down-regulation of proteins within the different groups. eq., equilibrium.

Deletion of *hfq* severely impacts *A. tumefaciens* fitness and virulence [31]. To reveal the molecular basis of this pleiotropic phenotype, we performed quantitative proteomics using isobaric tags for relative and absolute quantitation (iTRAQ). Almost half of all annotated *A. tumefaciens* proteins (2544 = 47.5%) were identified in three biological replicates of WT and Δ*hfq* cultures grown to stationary phase, indicating the high sensitivity of this method (Fig. 1A). Quantification of protein amounts was achieved by simultaneous LC-MS/MS analysis of the differently labeled peptides from WT and *hfq* mutant. Fold-changes (FC) from Δ*hfq*/WT ratios of all 2544 proteins were used to calculate a confidence interval of 95% (Fig. 1B). Given the calculated upper (FC 2.3) and lower (FC 0.45) bounds we found a total of 136 proteins, encoded from all four replicons, that were differentially abundant in the *hfq* mutant compared to the WT (Fig. 1B). 100 proteins were up-regulated, whereas 36 proteins were down-regulated (Table S4). Hfq-affected proteins were clustered into six physiological groups based on KEGG ontology. The major group comprises 71 proteins involved in transport mechanisms. Sixty-five of these proteins belong to the ABC transporter class (Fig. 1C). Further, 22 enzymes participating in metabolism of amino acids, carbohydrates, lipids or nucleotides, and involved in energy production/conversion were identified. While proteins from the transporter or enzyme class were mainly up-regulated in the *hfq* deletion strain (Fig. 1C, filled bars), proteins related to motility and chemotaxis were consistently down-regulated. Abundance of 13 proteins assigned to this group was reduced in the *hfq* mutant (e.g. Fla, FlaB, FliF, McpA, CheW). One protein involved in signal transduction and secretion mechanisms (TraG, conjugal transfer protein) and one protein associated with cell envelope biogenesis (Atu4877, short-chain dehydrogenase) were up-regulated in the Δ*hfq* strain. Twenty-eight hypothetical proteins with unknown function followed the overall trend of up-regulation in the *hfq* mutant.

66 of the Hfq-dependent proteins were (at least) pairwise encoded in 27 putative polycistronic operons (Fig. S2). Proteins matching the same operon were consistently up- or down- regulated. The fact that 23 of the identified operons encode at least one protein associated with ABC transporter uptake systems further supports a principal role of Hfq in nutrient acquisition.

### Establishment of a functional chromosomally encoded Hfq^3xFlag^ fusion

**Figure 2:**
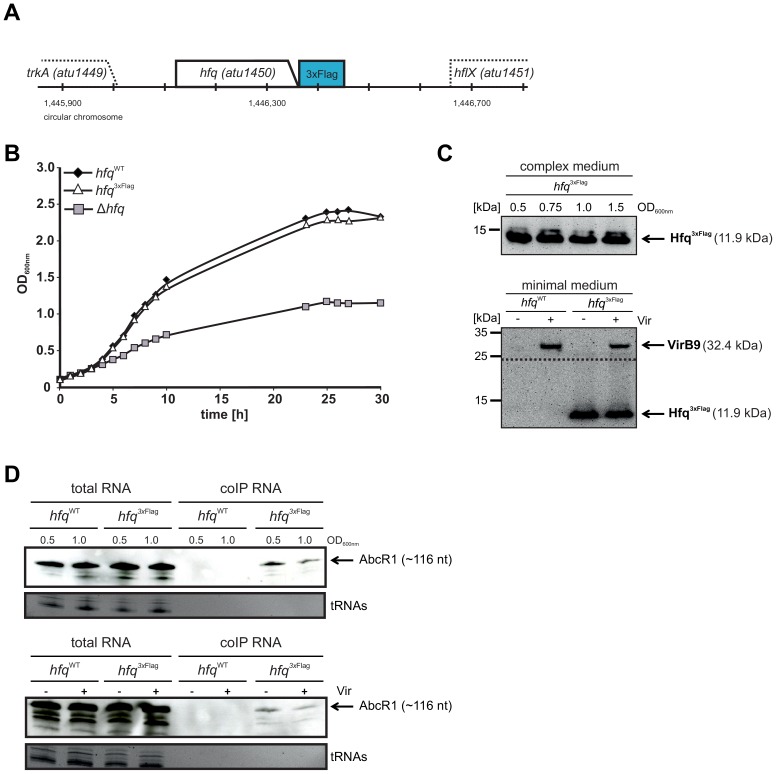
Construction of *hfq* ^3xFlag^ for co-immunoprecipitation. **A)** Chromosomal integration of a *3xFlag* sequence at the 3′ end of the *hfq* sequence. **B)** Growth experiments in complex medium confirmed functionality of the Hfq^3xFlag^ fusion. Growth of *hfq* ^WT^ and *hfq* ^3xFlag^ strains was comparable, while the Δ*hfq* mutant exhibited a severe growth defect. **C)** Western blot analysis of Hfq^3xFlag^ protein (∼11.9 kDa) from protein extracts isolated from *hfq* ^WT^ and *hfq* ^3xFlag^ strains. Proteins were isolated from exponential (OD 0.5) to stationary growth phase (OD 1.5). VirB9 was detected to confirm successful induction of virulence by addition of acetosyringone (+Vir). **D)** Purification of Hfq^3xFlag^ and isolation of co-purified RNA. Total RNA and co-immunoprecipitated RNA (coIP RNA) were analyzed by PAA gel electrophoresis and subsequent Northern blotting. AbcR1 was detected by a DIG-labeled RNA probe. Ethidium bromide stained tRNAs served as loading control. Equal amounts of coIP RNA were loaded, but no tRNAs were detectable in the corresponding lanes.

In a next step, we examined the Hfq regulon on the RNA level by using deep sequencing analysis of RNAs that were co-purified in a coIP with Hfq carrying a 3xFLAG epitope. The chromosomal *hfq* copy was replaced by a copy with a *3xFlag* sequence at the 3′ end (Fig. 2A). Whereas growth of the Δ*hfq* strain was severely reduced, growth of the *hfq* ^3xFlag^ strain was indistinguishable from *hfq* ^WT^ cultures, indicating that the C-terminal tagging did not interfere with its function (Fig. 2B). Hfq^3xFlag^ (∼11 kDa) protein amounts were comparable in all growth phases as verified by Western blot analysis using a monoclonal anti-Flag antibody (Fig. 2C, upper panel). Similar Hfq^3xFlag^ amounts were present when cells were grown in minimal medium at non-induced (−Vir) or virulence-induced (+Vir) conditions (Fig. 2C, lower panel). The T4SS protein VirB9 served as control for successful virulence induction and was only detected at +Vir conditions. All these experiment suggest that Hfq^3xFlag^ is functionally equivalent to the WT protein.

In the next step, we validated the suitability of Hfq^3xFlag^ to enrich Hfq-interacting RNAs. RNA was isolated from Hfq^3xFlag^ proteins in exponential (Exp), early stationary (Stat), non-induced (−Vir) and virulence-induced (+Vir) conditions. The known Hfq-dependent sRNA AbcR1 [31] was successfully recovered by Hfq^3xFlag^ as shown by Northern blot analysis (Fig. 2D). While AbcR1 was present in total RNA samples from *hfq* ^WT^ and *hfq* ^3xFlag^ cells, it was only found in samples containing the Flag-tagged protein after co-IP.

### Hfq-bound RNA pool varies in complexity and abundance

**Figure 3:**
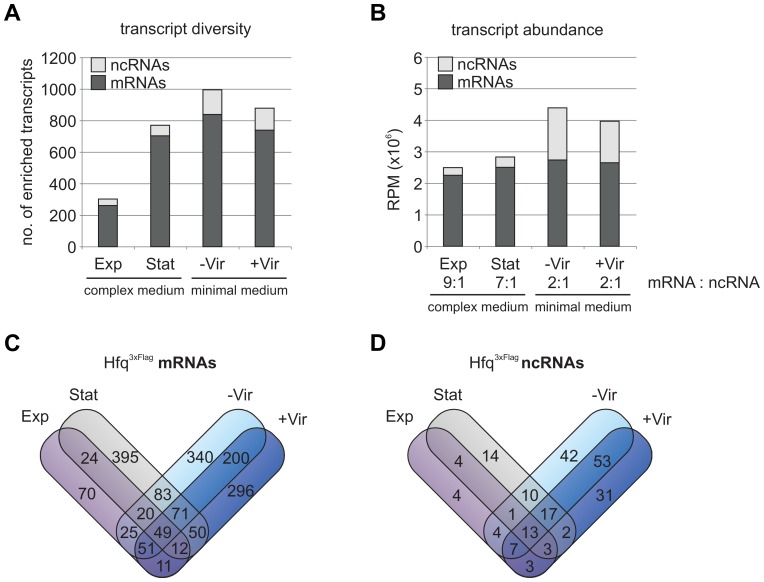
Hfq^3xFlag^ binds mRNAs and ncRNAs. **A)** Total numbers of mRNAs and ncRNAs enriched during Hfq^3xFlag^ coIP (transcript diversity) in Exp, Stat, -Vir and +Vir conditions. **B)** Abundance of mRNAs and ncRNAs enriched by Hfq^3xFlag^ (RPM) in the different growth phases. Ratio of mRNAs : ncRNA is indicated below the respective growth condition. Condition specific and overlapping enrichment of mRNAs **C)** and ncRNAs **D)** by Hfq^3xFlag^ at the different growth conditions. Exp, exponential; Stat, stationary; -Vir, non-induced; +Vir, virulence-induced; RPM, reads per million.

To identify Hfq-dependent RNAs, we performed RIP-seq experiments with *A. tumefaciens hfq* ^WT^ and *hfq* ^3xFlag^ strains grown to exponential (Exp) and stationary phase (Stat) in complex medium or at –Vir and +Vir conditions in minimal medium. Hfq^3xFlag^ protein was purified from cell extracts using DynaBeads with monoclonal anti-Flag antibody prior to RNA isolation, reverse transcription and cDNA sequencing. As expected, the number of reads from the Hfq^3xFlag^ libraries was generally higher (Table 1). RNAs enriched at least 2-fold (Hfq^3xFlag^/Hfq^WT^) with a minimal raw read count (RRC) of 50 in the Hfq^3xFlag^ libraries were considered Hfq bound. 1906 different RNAs were enriched, including 1697 mRNAs, 208 ncRNAs and one tRNA (tRNA-Gly, *atu0435*). Despite the rather large number of Hfq-bound mRNAs, many known targets were enriched about 2-fold (e.g. *atu2422*, 2.61-fold; *atu4678*, 2.2-fold; *malE*, 2.24-fold; *atu4113*, 2.3-fold) [31], indicating sufficient specificity of the observed interaction with Hfq. The transcript diversity of mRNAs and ncRNAs differed notably between Exp (262 mRNAs/39 ncRNAs), Stat (704 mRNAs/64 ncRNAs), −Vir (839 mRNAs/147 ncRNAs) and +Vir (740 mRNAs/129 ncRNAs) conditions (Fig. 3A). The highest number of different Hfq-associated RNAs was found during stress conditions (Stat growth, −/+ Vir).

**Table 1:** Total number of uniquely aligned reads.

		growth condition			
		exponential (Exp)	stationary (Stat)	non-induced (−Vir)	virulence-induced (+Vir)
total number of uniquely aligned reads	*hfq* ^WT^	5,490,606	4,429,841	265,804	1,489,476
	*hfq* ^3xFlag^	8,230,008	4,652,974	9,374,442	8,199,109

Reads per million (RPM, raw read counting per gene divided by total number of aligned reads×10^6^) from mRNAs and sRNAs enriched at the different conditions were summed up, to describe the actual load of RNAs on Hfq under different conditions (Fig. 3B). In complex medium, 70 to 90% of all Hfq-trapped RNAs were mRNAs. The relative number of Hfq-bound ncRNAs was highest in minimal medium suggesting that RNA-mediated regulation plays an important role when nutrients are scarce. A large number of transcripts (1101 mRNAs/91 ncRNAs) were specifically enriched at only one condition (Fig. 3C, 3D). The remaining RNAs were found in at least two conditions (547 mRNAs/104 ncRNAs) or in all conditions (49 mRNAs/13 ncRNAs). Although no Hfq-independent RNAs were described in *A. tumefaciens* so far, the well-known housekeeping RNAs 6S, tmRNA and SRP were not enriched by Hfq^3xFlag^ (Fig. S3). These observations are comparable to results from various other Hfq-RNA pull-down experiments [9], [11], [58], [59], further supporting specificity of the observed RNA-binding to Hfq^3xFlag^ in our study.

### Hfq primarily influences nutrient acquisition and cellular metabolism

**Figure 4:**
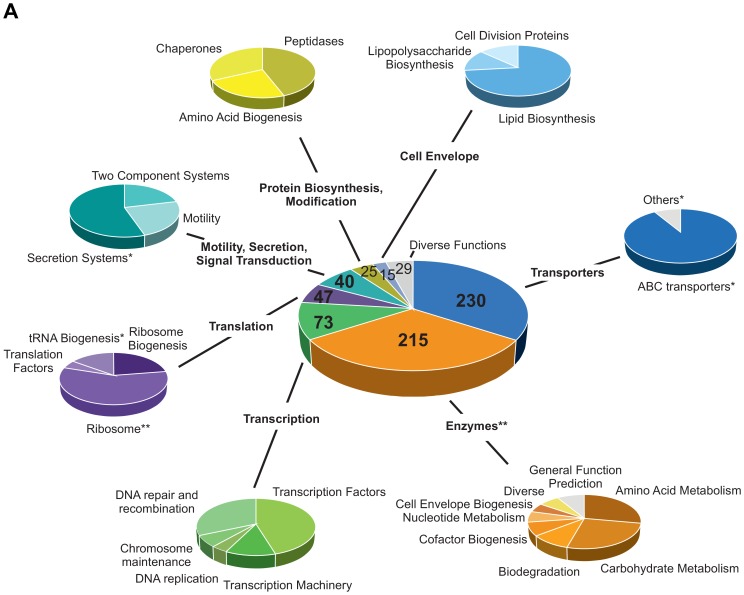
mRNAs enriched by Hfq^3xFlag^ cluster into physiological relevant groups. Clustering of Hfq^3xFlag^ enriched mRNAs into seven physiological coherent groups by KEGG ontology. Main groups are subdivided to specify the biological function of the respective proteins. Statistical relevance: * p-value <0.05, ** p-value <0.01.

The 1697 mRNAs enriched by Hfq^3xFlag^ represent 31% of the coding potential of *A. tumefaciens* (Table S5). Seventy-four mRNAs enriched by Hfq encode proteins that were differently abundant during iTRAQ analysis, strongly indicating post-transcriptional regulation. A large proportion (58%) of the Hfq-bound mRNAs encodes proteins of unknown function and was excluded from functional analysis. The remaining 721 mRNAs were clustered according to KEGG ontology into seven main groups ((1) transporters, (2) enzymes, (3) transcription, (4) translation, (5) motility, secretion and signal transduction, (6) protein biosynthesis and modification and (7) cell envelope biogenesis), each subdivided into several subgroups (Fig. 4). Due to the high number of enriched transcripts, statistical relevance (p-value <0.05) of the performed clustering was partly limited. Yet, the most prominent groups enriched during coIP correlate with the results obtained by mass spectrometry. Consistent with the observations from our proteome analysis, the largest group of 230 mRNAs enriched by Hfq was associated with transport processes. The vast majority (95%) encodes proteins from the ABC transporter class and was significantly overrepresented in the Hfq^3xFlag^ samples (p<0.05). 215 mRNAs encode enzymes participating in various cellular processes e.g. amino acid and carbohydrate metabolism, biodegradation of molecules, and cofactor and nucleotide biogenesis. Quite interestingly, Hfq bound 73 mRNAs encoding proteins involved in transcription. About one half of the corresponding proteins were transcription factors. Therefore, indirect influence of transcription by regulation of transcription factors might be an additional layer of Hfq-mediated regulation. The other half of proteins was involved in the transcription process itself, e.g. transcription machinery, DNA replication and DNA maintenance. Although ribosomal RNAs were not depleted from Hfq^3xFlag^ enriched RNA pools, neither rRNAs nor tRNAs (except for tRNA-Gly) were specifically enriched. Still, Hfq^3xFlag^ bound 23 mRNAs encoding ribosomal proteins from both small and large ribosomal subunits as well as RNases (RNaseP, RNaseE) involved in ribosome biogenesis.

mRNAs enriched and assigned to motility, secretion and signal transduction partly encode the VirB/D4 type IV secretion system, including *virB1*, *B5*, *B6*, *B7*, *B8*, *B10*, *B11* and mRNAs directly associated with *A. tumefaciens*-specific DNA transfer and virulence like *virC1*, *C2*, *virD1*, *D3*, *D5* and *virE1*-*3.* The *virC2* and *virE3* mRNAs were enriched in –Vir and +Vir conditions, whereas the other *vir*-mRNAs were exclusively enriched at +Vir conditions. Additional mRNAs encoding motility (e.g. FlaF, FlgL) and chemotaxis-associated proteins (e.g. McpA, CheD) were enriched in accordance with Hfq-dependent changes in protein level already observed by iTRAQ analysis. Protein biosynthesis and modification was also associated with Hfq. Several mRNAs encoding chaperones (e.g. HslV), peptidases (e.g. protease ClpP) or proteins involved in amino acid biogenesis (e.g. AtrB, HemA) were Hfq-bound. Interestingly, the *hfq* ^3xFlag^ mRNA was also enriched by Hfq^3xFlag^ in -Vir (3.8-fold) and +Vir (2.6-fold) conditions (Fig. S4). A rather small group of mRNAs encodes proteins participating in cell envelope biogenesis, lipid and lipopolysaccharide biosynthesis (e.g. KdtA, protease) as well as cell division (e.g. MinD).

### Identification and validation of Hfq-dependent ncRNAs

**Figure 5:**
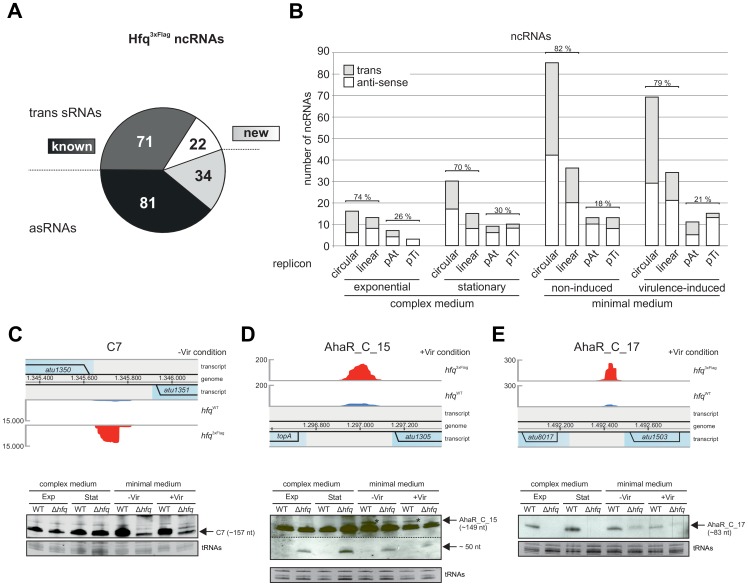
Identification of Hfq-dependent ncRNAs. **A)** Numbers of asRNAs and trans sRNAs enriched by Hfq^3xFlag^. 152 known and 56 new ncRNAs were enriched. **B)** Expression of ncRNAs during the different growth phases from the four *A. tumefaciens* replicons. Relative expression from chromosomes (circular, linear) and mega-plasmids (At, Ti) at the different conditions is indicated in %. Northern blots of RNA isolated from WT and Δ*hfq* strains validated Hfq-dependency of trans encoded sRNA **C)** C7 and the newly identified sRNAs **D)** AhaR_C_15 and **E)** AhaR_C_17 (lower panel). Expression locus and reads from the genome browser are indicated on top. Ethidium bromide stained tRNAs served as loading control. Exp, exponential; Stat, stationary; -Vir, non-induced; +Vir, virulence-induced.

Out of the 208 Hfq^3xFlag^ enriched regulatory ncRNAs (Table S6), 152 have been described in previous studies [35], [36]. AbcR1 was strongly enriched under –Vir (33870 RPM, 5-fold) and +Vir (31705 RPM, 27-fold) conditions, confirming specific enrichment of Hfq-dependent RNAs. 56 ncRNAs were newly discovered in our study (Fig. 5A). The pool of ncRNAs enriched by Hfq^3xFlag^ was comprised of 93 trans-encoded sRNAs and 115 asRNAs with partial or complete complementarity to the target transcript encoded on the complementary strand. The ncRNAs were transcribed from all 4 replicons of the *A. tumefaciens* genome (Fig. 5B), and the distribution largely corresponded to the respective size of the replicons (Fig. S5). Both circular and linear chromosomes constitute 87% of the genome and harbor 70–80% of the ncRNAs, only slightly varying between the different conditions (Fig. 5B). In general, the number of ncRNAs was much higher in minimal medium than in complex medium.

To investigate Hfq-dependency of selected Hfq^3xFlag^ enriched ncRNAs, Northern blot experiments with total RNA isolated from WT and Δ*hfq* strains were performed. The three strongly enriched trans-encoded sRNAs C7, AhaR_C_15, AhaR_C_17 were chosen (Fig. 5C–E). C7 is encoded between *atu1350* and *atu1351* on the (−) strand and was 20-fold enriched in minimal medium (Fig. 5C, upper panel). The C7 RNA encoded on the circular chromosome has previously been identified in a screen for ncRNAs [35]. The amounts of C7 were comparable in complex medium. An influence of Hfq was apparent in minimal medium when C7 levels were reduced in the Δ*hfq* strain (Fig. 5C, lower panel). The two newly identified ncRNAs AhaR_C_15 and AhaR_C_17 were also affected by Hfq. AhaR_C_15 is transcribed from the (+) strand between *topA* and *atu1305* and was enriched 6.6-fold during coIP of the +Vir condition (Fig. 5D, upper panel). The sRNA full length signal was slightly larger than a non-identified signal present in all conditions. The RNA was barely detectable in Northern blots of total RNA from Exp and Stat growth phases (Fig. 5D, lower panel). In -Vir and +Vir conditions regulation was more evident, reflected by reduced amounts of the sRNA in the *hfq* mutant. In addition, a small fragment of about 50 nucleotides was detected in the Δ*hfq* deletion strain suggesting processing of AhaR_C_15. The AhaR_C_17 sRNA is encoded between *atu8017* and *atu1503* from the (+) strand and was enriched 10.3-fold in the coIP of the +Vir condition (Fig. 5E, upper panel). Northern blot analysis demonstrated a clear Hfq-dependence of the sRNA transcript under all tested conditions (Fig. 5E, lower panel).

### Hfq influences asRNAs and target mRNAs

**Figure 6:**
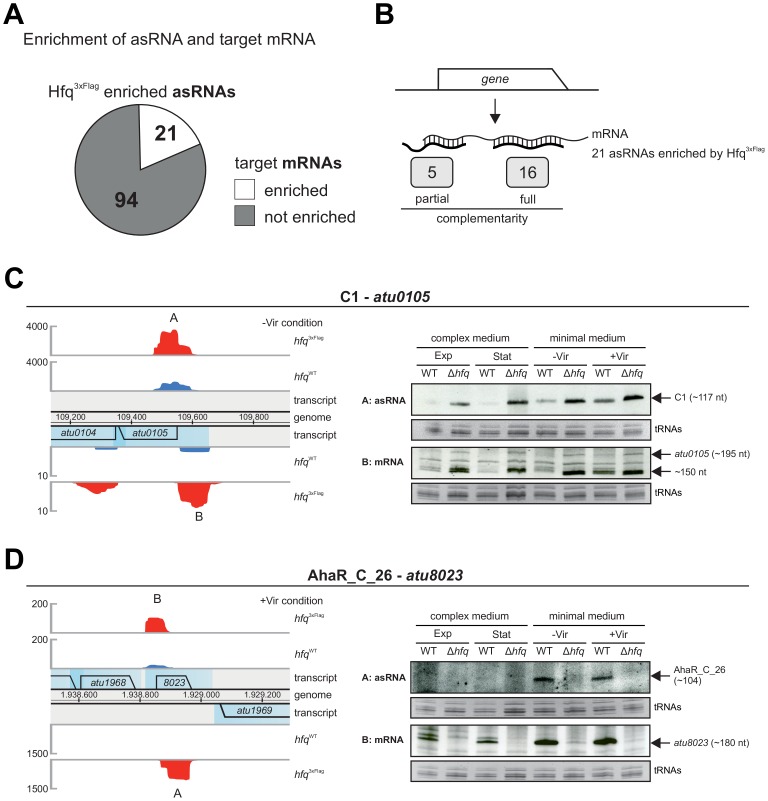
Hfq binds asRNAs and their cognate target mRNAs. **A)** 21 of the 115 asRNAs were enriched simultaneously with their target mRNA encoded on the complementary strand. **B)** Complementarity of the 21 asRNA-mRNA pairs. 16 asRNAs were fully complementary to their designated target mRNAs. **C)**, **D)** Northern blot analysis of asRNAs and target mRNAs with full complementarity. Location and mapped reads of **C)** C1 and *atu0105* and **D)** AhaR_C_26 and *atu8023,* are indicated by the genome browser view (left). Northern blot analysis of RNA isolated from WT and Δ*hfq* strains grown under different conditions (right). Ethidium bromide stained tRNAs or 16S rRNAs served as loading control. Exp, exponential; Stat, stationary; -Vir, non-induced; +Vir, virulence-induced.

Out of the 115 asRNAs enriched by Hfq^3xFlag^, 21 asRNAs were enriched simultaneously with their cognate mRNA target encoded on the complementary strand (Fig. 6A). Sixteen of these Hfq-associated asRNA-target mRNA pairs are fully complementary (Fig. 6B, Table S7) and thus would normally be expected to not require the aid of Hfq. Northern blot analysis with two selected asRNAs and their putative target mRNAs, however, confirmed a clear influence of Hfq on both interaction partners.

The asRNAs C1 (3.6-fold) and AhaR_C_26 (24.8-fold) were enriched with their designated target mRNAs *atu0105* (2.9-fold) and *atu8023* (6.4-fold), respectively (Fig. 6C, D). C1 and *atu0105* were enriched in -Vir conditions (Fig. 6C, left) and abundance was consistently higher in the Δ*hfq* strain in all tested conditions compared to WT levels (Fig. 6C, right). The same was true for the sense RNA *atu0105*. AhaR_C_26 and *atu8023* were enriched in +Vir conditions (Fig. 6D). Consistent with low read numbers AhaR_C_26 was barely detectable on Northern blots. Nonetheless, both AhaR_C_26 and its target mRNA *atu8023* were notably less abundant in the Δ*hfq* mutant strain at all conditions.

## Discussion

### Hfq, a global mediator of nutrient uptake, metabolism and motility

**Figure 7:**
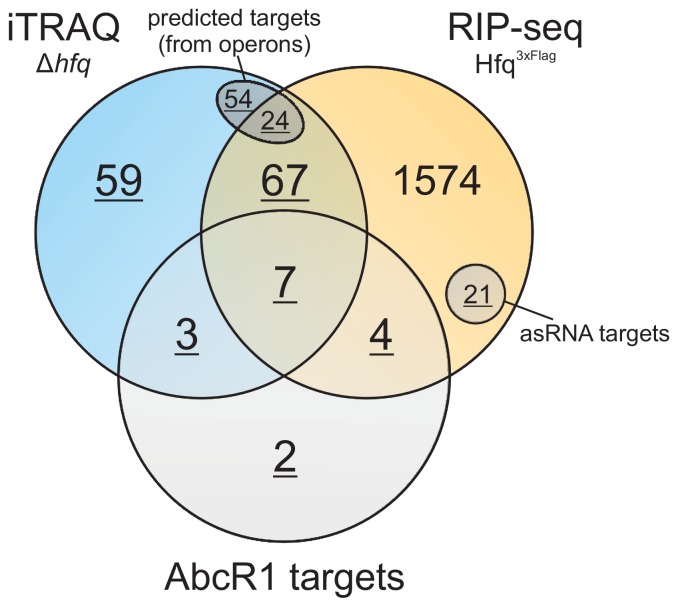
Hfq regulon in *A. tumefaciens.* Summary of mRNAs/proteins influenced by Hfq in *A. tumefaciens*. Results from iTRAQ and RIP-seq (this study) and AbcR1 targets [37], [38] were combined. Operons encoding at least 2 equally regulated proteins (iTRAQ) were included and all encoded proteins were assumed to be Hfq-dependent. 21 target mRNAs simultaneously enriched with an asRNA were also included. Hfq-dependent proteins/mRNAs used for network predictions are underlined (Fig. 8).

In this study, we combined two global approaches targeted at the identification of Hfq-affected proteins (iTRAQ) and Hfq-associated RNAs (RIP-seq) to further our understanding of the fundamental role of the RNA chaperone in *Agrobacterium* physiology. Our results reveal a huge Hfq regulon (Fig. 7) and extend it far beyond the eight previously identified Hfq-dependent genes and proteins in *A. tumefaciens* [31], [38].

The abundance of a large number of ABC transporters (71), enzymes (22) and motility related proteins (13) was significantly altered in the *hfq* mutant. As one would expect, physiologically related proteins were consistently up or down-regulated, partly caused by coupled expression from polycistronic transcripts (Fig. S2). Most of the proteins (73%) were repressed depending on Hfq, including ABC transporters for oligopeptide (DppA), proline/glycine betaine (ProX), amino acid (EhuB), putrescine (PotF), maltose (MalE) and nopaline (NocT) import. 13 ABC transporter proteins identified in our combined study, among them MalE, NocT, Atu2422, were previously reported to be negatively regulated by AbcR1 (Fig. 7) and were therefore overrepresented in the *hfq* mutant [37], [38] (Table S4). Whether more of the regulated ABC transporters are also controlled by AbcR1 or other sRNAs as demonstrated in various organisms [60], [61], [62], [63], [64] remains to be shown. *A. tumefaciens* encodes approx. 150 ABC transporters [33] and it is evident that precise regulation of these systems is necessary in the competitive rhizosphere. Unregulated permanent production of all 150 import systems would impose a costly metabolic burden [65]. Rapid translational control by Hfq-dependent sRNAs (e.g. AbcR1) helps to adjust the transporter repertoire in response to the metabolic demand and maintains bacterial fitness.

Apart from nutrient uptake, 13 proteins associated with motility were consistently down-regulated. FliF (MS-ring), FlgB (rod), FlgE (hook), Fla and FlaB (filament) are structural components of the flagellum, enabling rotation and functionality [66], [67]. Misregulation of motility-associated proteins upon *hfq* deletion in other bacteria resulted in reduced motility, independent of whether they were up-regulated (*Serratia* sp. ATCC 39006) or down-regulated (*S. meliloti*) [68], [69]. Our results are in line with previously reported motility defects in the *A. tumefaciens* Δ*hfq* strain [31]. Hfq-binding of the *fliL* mRNA as part of the *flgB* encoding polycistronic operon supports translational regulation of at least some motility-associated transcripts. Whether all of the affected proteins identified in our study are directly influenced by the RNA chaperone or alterations result from an indirect response to the *hfq* deletion, needs to be verified in further experiments.

### Hfq bound mRNAs and ncRNAs – a layout of physiological state

RIP-seq of *A. tumefaciens* Hfq-bound RNAs from different growth phases revealed an extensive RNA binding potential of the RNA chaperone. 1697 mRNAs and 209 ncRNAs were enriched. The diversity of the mRNA and ncRNA pools varied notably between the tested conditions (Fig. 3). Under nutrient-limited conditions (minimal medium) the diversity of Hfq-enriched mRNAs and the relative amount of ncRNAs increased substantially. Condition specific binding to Hfq might give first hints about the physiological function of those ncRNAs. Hfq-mediated adaptation to stress is common in bacteria [11], [12], [25], [59], [70], [71], [72], [73]. Riboregulation is less expensive than protein-mediated regulation since ncRNAs are shorter than most mRNAs and do not require translation [1]. This may explain the dramatic shift in the Hfq-associated mRNA:ncRNA population from 9∶1 to 2∶1 in response to nutrient limitation. Some regulatory sRNAs also encode small peptides, e.g. SR1 from *Bacillus subtilis* [74]. Determining potential protein-encoding ncRNAs identified in our study will be a challenging question for future studies.

Although *E. coli* Hfq was shown to bind tRNAs *in vitro*, our data on *A. tumefaciens* Hfq are consistent with reports on *Salmonella enterica* and *S. meliloti* Hfq showing specificity towards mRNAs and ncRNAs and reveal only tRNA-Gly associated with Hfq [10], [59], [75]. The regulatory mechanism of tRNA-Gly regulation and its physiological role are yet to be determined. It is conceivable, that tRNA-Gly enrichment is indirectly mediated by co-binding with another Hfq-interacting RNA. In *Bacillus subtilis,* T-Box riboswitches bind uncharged tRNAs and are also associated with Hfq [72]. So far, T-Box riboswitches were mostly known from Gram-positive bacteria, but have been recently found in Gram-negative bacteria as well [76], [77]. A ternary complex of Hfq-riboswitch-tRNA could explain specific enrichment of a single tRNA species despite high homology in structure and sequence of this molecule class.

Although Hfq does not directly bind rRNAs, Hfq seems to play a role in ribosome biogenesis since we found 23 mRNAs encoding ribosomal proteins (13 L-proteins, 10 S-proteins) associated with Hfq. The *rpsE* mRNA was already shown to be Hfq-dependent [31], and binding of the co-transcribed *rplN*, *rpmC*, *rplP*, *rplC* and *rpsC* mRNAs further supports translational regulation of ribosome biogenesis by Hfq. Hfq-specific binding of mRNAs encoding ribosomal proteins seems to be widespread as it was also observed in *E. coli*, *R. sphaeroides* and *S. meliloti* [11], [59], [78]. Whether these transcripts are influenced by Hfq and/or underlie ncRNA-mediated regulation is a promising issue for future research.

### Hfq binding to antisense RNAs

Among the 209 Hfq-associated ncRNAs identified by RIP-Seq, 56 ncRNAs were not found in previous studies [35], [36]. They add to the growing list of *A. tumefaciens* ncRNAs and extend it to a total of 677. It seems that Hfq plays an important role in antisense regulation since 115 asRNAs were specifically enriched by the RNA chaperone. Strikingly, 21 asRNAs were enriched along with their cognate target mRNA derived from the complementary strand and we confirmed Hfq-dependent regulation of two asRNA-mRNA pairs (Fig. 6). asRNAs have rarely been found associated with Hfq [9], [11], [12], [72]. This and the fact that asRNAs and target mRNAs are transcribed in close spacial proximity and by definition share perfect sequence complementarity, led to a plausible model concluding that Hfq is dispensable for cis-encoded antisense RNA regulation [13]. Although this may be true for many bacteria, asRNA-regulation was already described for the *E. coli* Tn*10*/IS*10* system (RNA-IN, RNA-OUT) [79], and genomic SELEX identified preferential binding of Hfq to AU-rich sequences antisense of protein coding genes [80]. Additional Hfq coIP experiments revealed binding of 67 asRNAs to the RNA chaperone [81]. Our study in concert with a recent Hfq-coIP study in *S. meliloti* [59], adds further evidence to the importance of Hfq-associated antisense transcripts in translational regulation.

### Hfq contributes to *A. tumefaciens* virulence

An *A. tumefaciens hfq* mutant is severely impaired in tumor formation on plants [31]. Similar virulence defects have been reported for several other pathogens [32], [73], [82], [83], [84], [85], [86]. RIP-seq identified 296 mRNAs and 31 ncRNAs specifically enriched by Hfq under virulence-induced (+Vir) conditions in *A. tumefaciens*. Importantly, virulence-related mRNAs of the *virB*, *virC* and *virE* operons were associated with Hfq. Among those, the *virB1* (5,467 RPM, 3-fold) and *virE3* (13,770 RPM, 6.7-fold) mRNAs were enriched most explicitly. The *virB* and *virE* mRNAs are among the most highly induced transcripts under virulence conditions [87]. Yet, protein abundance of the *virB* operon encoded VirB2, B5, B8 and B9 proteins was not significantly affected in the *hfq* mutant [31] and Northern blot analysis with a *virE3* specific probe did not reveal obvious changes in RNA amounts in the Δ*hfq* strain as compared to WT levels. The identification of 31 ncRNAs specifically enriched under virulence conditions suggests a substantial regulatory potential. Further, regulation of the TraR anti-activator TraM (identified by iTRAQ) might also contribute to efficient infection. TraM (Atu6131) was down-regulated in absence of Hfq, while the conjugal transfer coupling protein TraG (Atu6124) was up-regulated. TraR is a transcriptional regulator and a key protein in replication and conjugation of the Ti-plasmid, directly linked to quorum-sensing and virulence [88]. TraR activates expression of the *tra*-operon including *traG*. In absence of Hfq, TraM amounts decrease, releasing TraR inhibition and resulting in an increase of TraG. Therefore, by influencing TraM, Hfq might contribute to Ti-plasmid replication and conjugation, thus modulating infection efficiency.

### The *A. tumefaciens* Hfq core regulon

**Figure 8:**
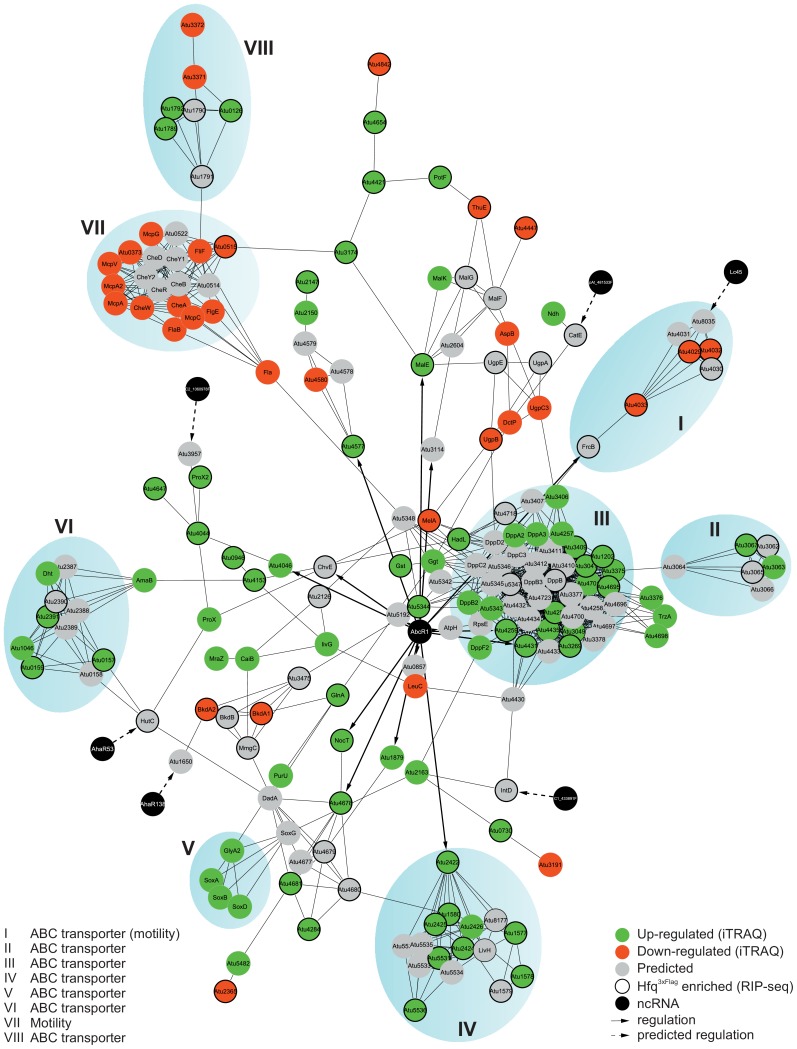
Model of the Hfq core regulon. All 241 proteins associated with Hfq regulation (Fig. 7, underlined) were checked for putative interactions by String 9.1 software. Visualization of the resulting network was performed using Cytoscape 3.1.0. 207 proteins were interconnected and 7 ncRNAs (black) with predicted (dashed line) or validated (continuous line) target mRNAs inside the network were added. Proteins identified by iTRAQ were marked when up-regulated (green), down-regulated (red) or predicted to be influenced (grey). Corresponding mRNAs identified during Hfq^3xFlag^ coIP were indicated by bold circles. Hfq or AbcR1-dependent mRNAs validated by Northern blots in prior studies [37], [38] were indicated by asterisks. Striking clustering of interacting nodes was marked by blue spheres and physiological functions were assigned according to protein functions of the involved proteins.

Our study places Hfq in the center of a complex posttranscriptional network that has a profound impact on the *A. tumefaciens* transcriptome and proteome. In order to visualize putative connections between Hfq-dependent proteins, we used the String 9.1 webserver [55] to predict physical and physiological interactions of the 241 proteins with known (or presumed) Hfq-dependent regulation highlighted in Fig. 7 (underlined). By this, we assembled a comprehensive network connecting 197 proteins (44 proteins did not connect to the main regulon) (Fig. 8). Since the network includes 6 asRNA targets, we manually included the corresponding asRNAs and the global regulator AbcR1 (black) with validated (continuous line) or predicted (dashed line) regulation of their targets. Interconnection of most of the Hfq-dependent proteins highlights efficient regulation of whole physiological circuits (blue shaded). The impact of Hfq on the *A. tumefaciens* proteome already indicated uniform regulation of polycistronic operons. Strikingly, assembly of the Hfq-regulon demonstrates regulation of nutrient uptake and motility beyond influence on single proteins or transport systems. Mainly ABC transporters (II, III, IV, V, VI, VIII) but also motility and chemotaxis related proteins (I, VII) are integrated into a complex intertwined network. Multilayered regulation is further supported by two findings. First, 34 of the Hfq-enriched mRNAs encode transcriptional regulators, typically controlling transcription of multiple genes. Second, Hfq binds its own mRNA suggesting auto-regulatory control as in *E. coli* or *S. meliloti* (Fig. S3) [11], [89], [90].

In summary, our data show a fundamental role of Hfq in the genome-wide regulation of nutrient uptake, metabolism and motility in *A. tumefaciens*. Hfq-mediated riboregulation is not restricted to partially complementary sRNA-mRNA interactions but includes interactions between fully complementary antisense and messenger RNAs. More than 30 ncRNAs were associated with Hfq under virulence conditions suggesting that the RNA chaperone may be of prime importance in bacteria-plant interaction.

## Supporting Information

Figure S1 **Categorization of transcripts for RIP-seq quantification.**
(TIFF)

Figure S2 **Operon predictions.**
(TIFF)

Figure S3 **Housekeeping RNAs were not enriched by Hfq^3xFlag^.**
(TIFF)

Figure S4 ***hfq*** **mRNA bound by Hfq.**
(TIFF)

Figure S5 **Relative size of the** ***A. tumefaciens*** **replicons.**
(TIFF)

Table S1 **Bacterial strains and plasmids used in this study.**
(XLSX)

Table S2 **Oligonucleotides used in this study.**
(XLSX)

Table S3 **Mapping statistics from RNA sequencing data analysis.**
(XLSX)

Table S4 **Proteins differentially abundant in** ***A. tumefaciens*** **Δ** ***hfq*** **(iTRAQ).**
(XLSX)

Table S5 **mRNAs enriched by Hfq^3xFlag^.**
(XLSX)

Table S6 **ncRNAs enriched by Hfq^3xFlag^.**
(XLSX)

Table S7 **asRNA and mRNA target-enrichment by Hfq^3xFlag^.**
(XLSX)
