# Supplementary figures and images for: Profound Impact of Hfq on Nutrient Acquisition, Metabolism and Motility in the Plant Pathogen Agrobacterium tumefaciens
Source: PLoS One. 2014 Oct 17;9(10):e110427. doi: 10.1371/journal.pone.0110427 (PMC4201532; doi:10.1371/journal.pone.0110427)

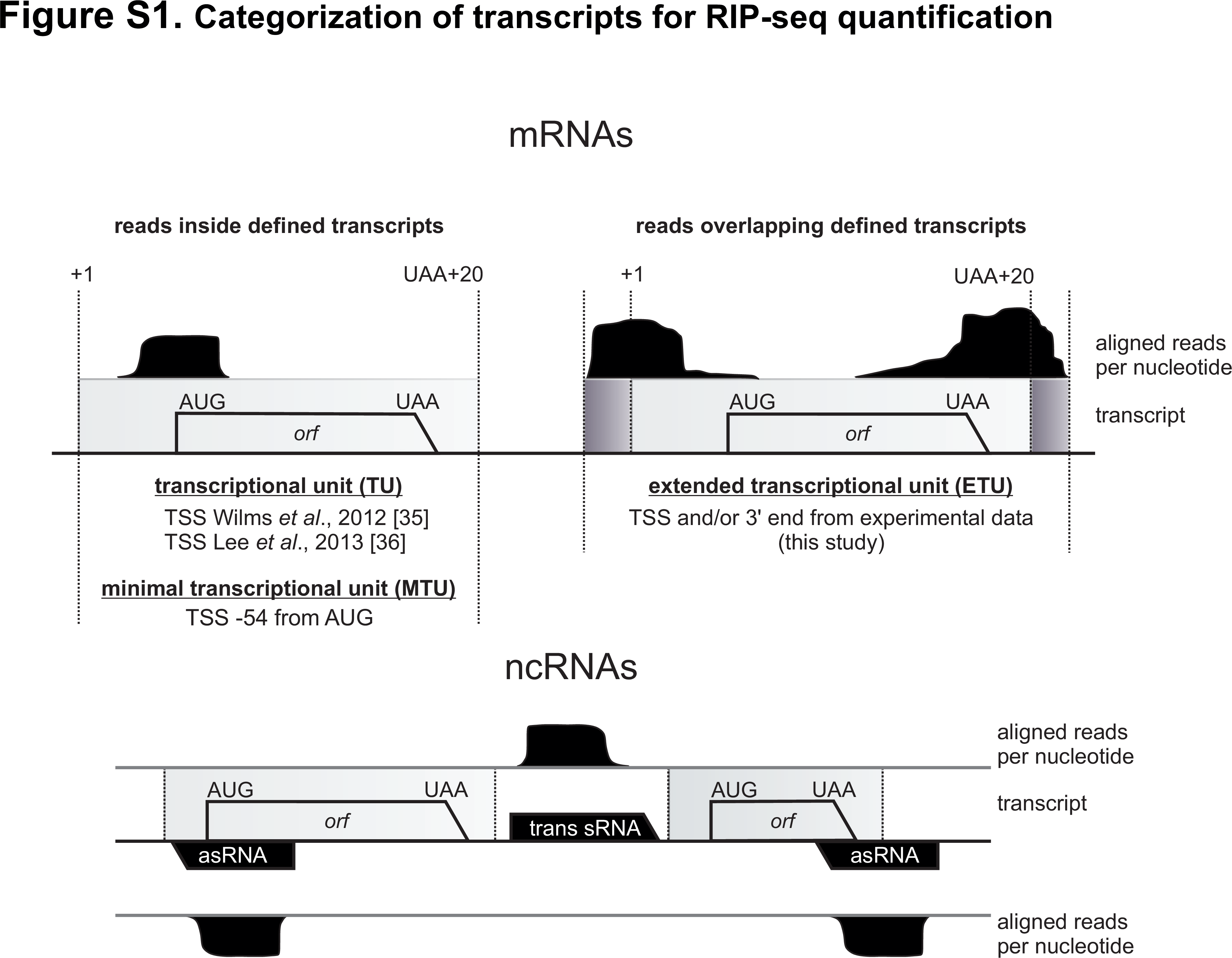

Supplement: Figure S1 — Categorization of transcripts for RIP-seq quantification. (TIFF) [file pone.0110427.s001.tiff]

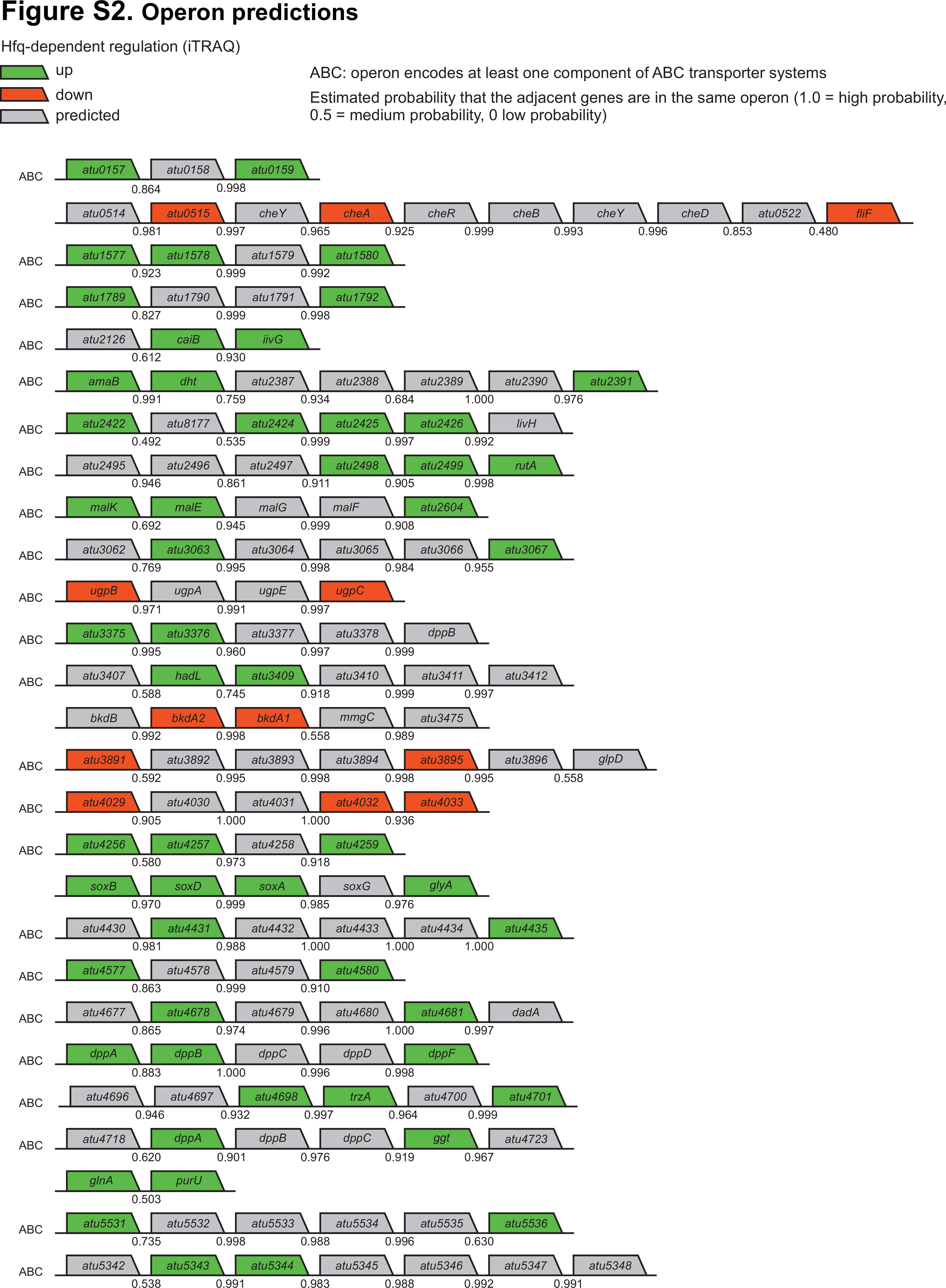

Supplement: Figure S2 — Operon predictions. (TIFF) [file pone.0110427.s002.tiff]

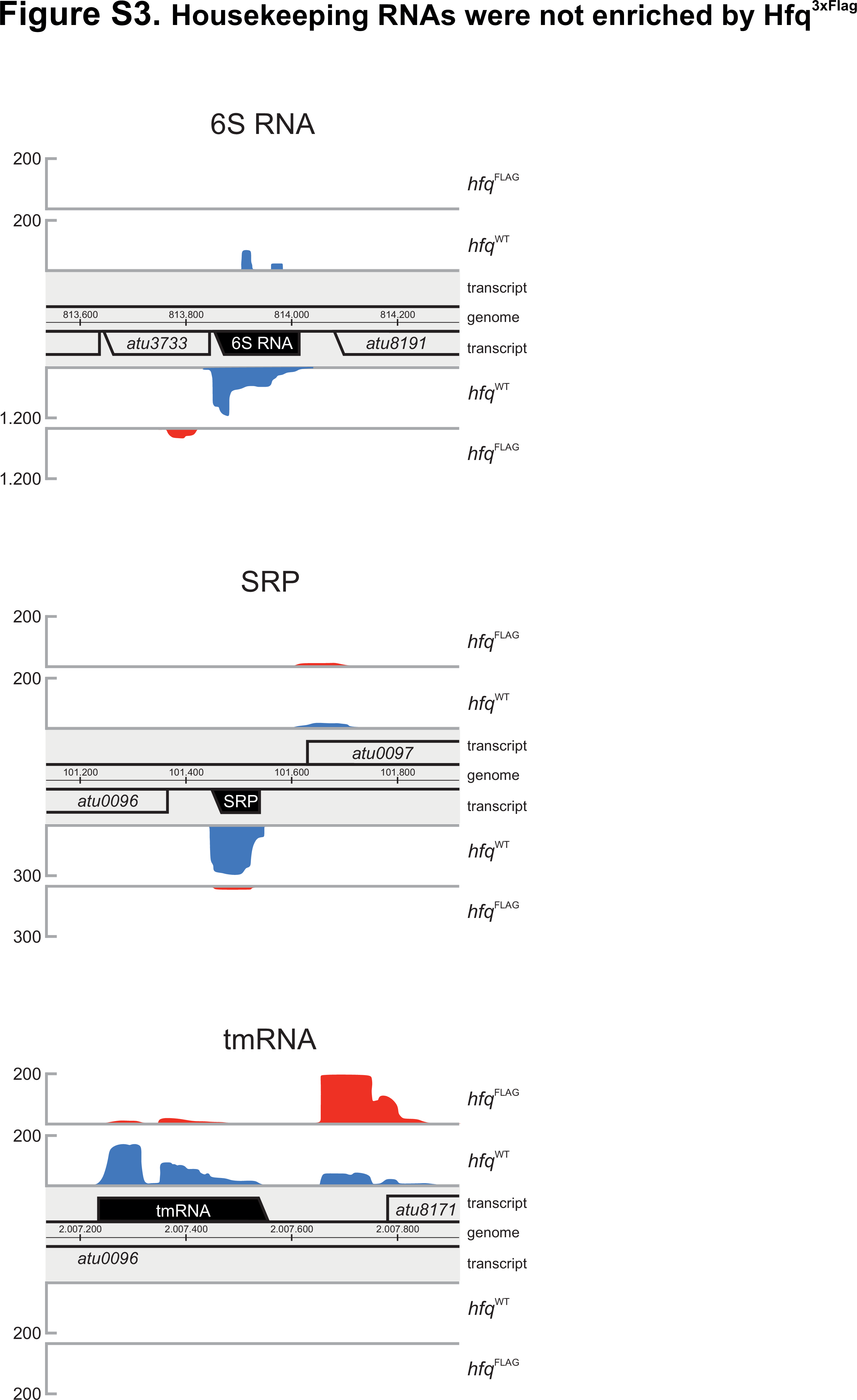

Supplement: Figure S3 — Housekeeping RNAs were not enriched by Hfq3xFlag. (TIFF) [file pone.0110427.s003.tiff]

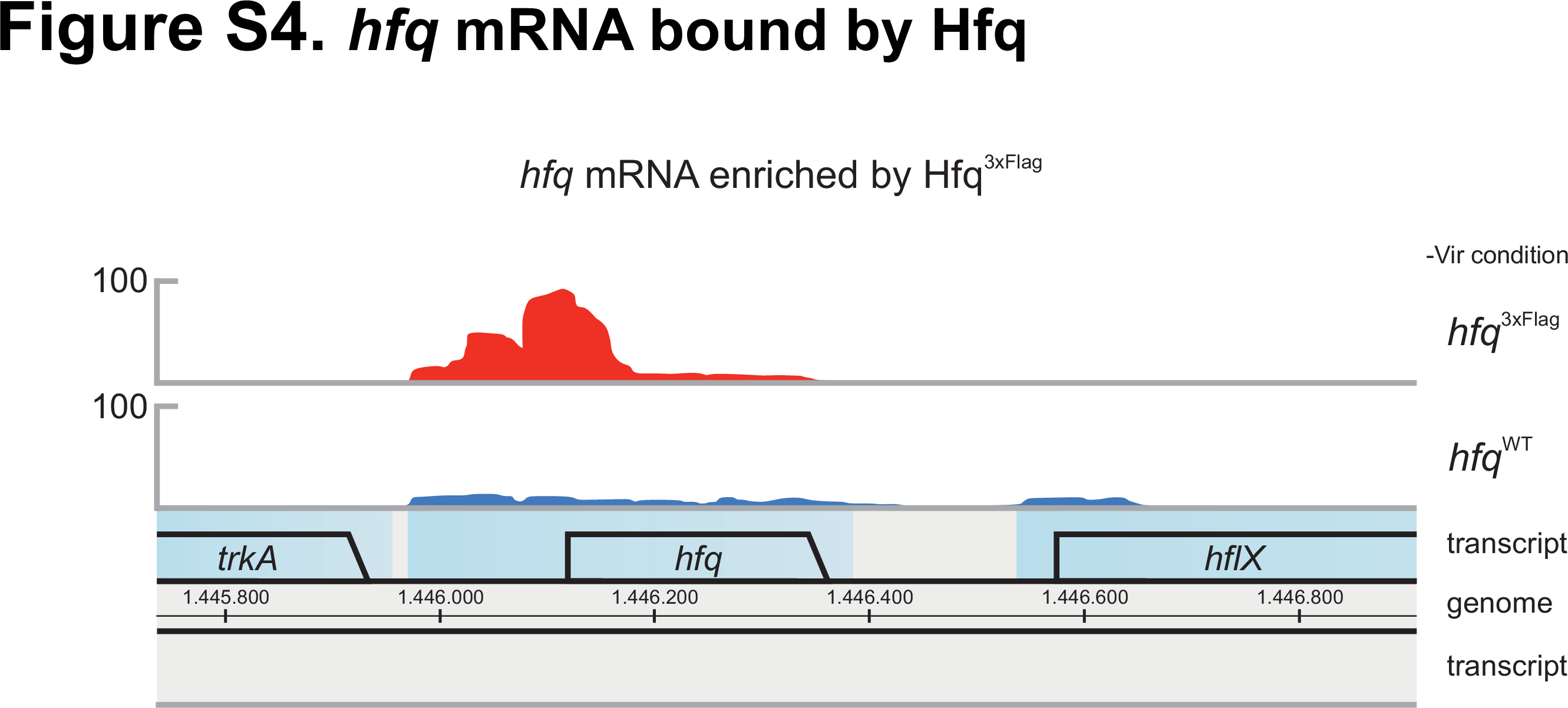

Supplement: Figure S4 — hfq mRNA bound by Hfq. (TIFF) [file pone.0110427.s004.tiff]

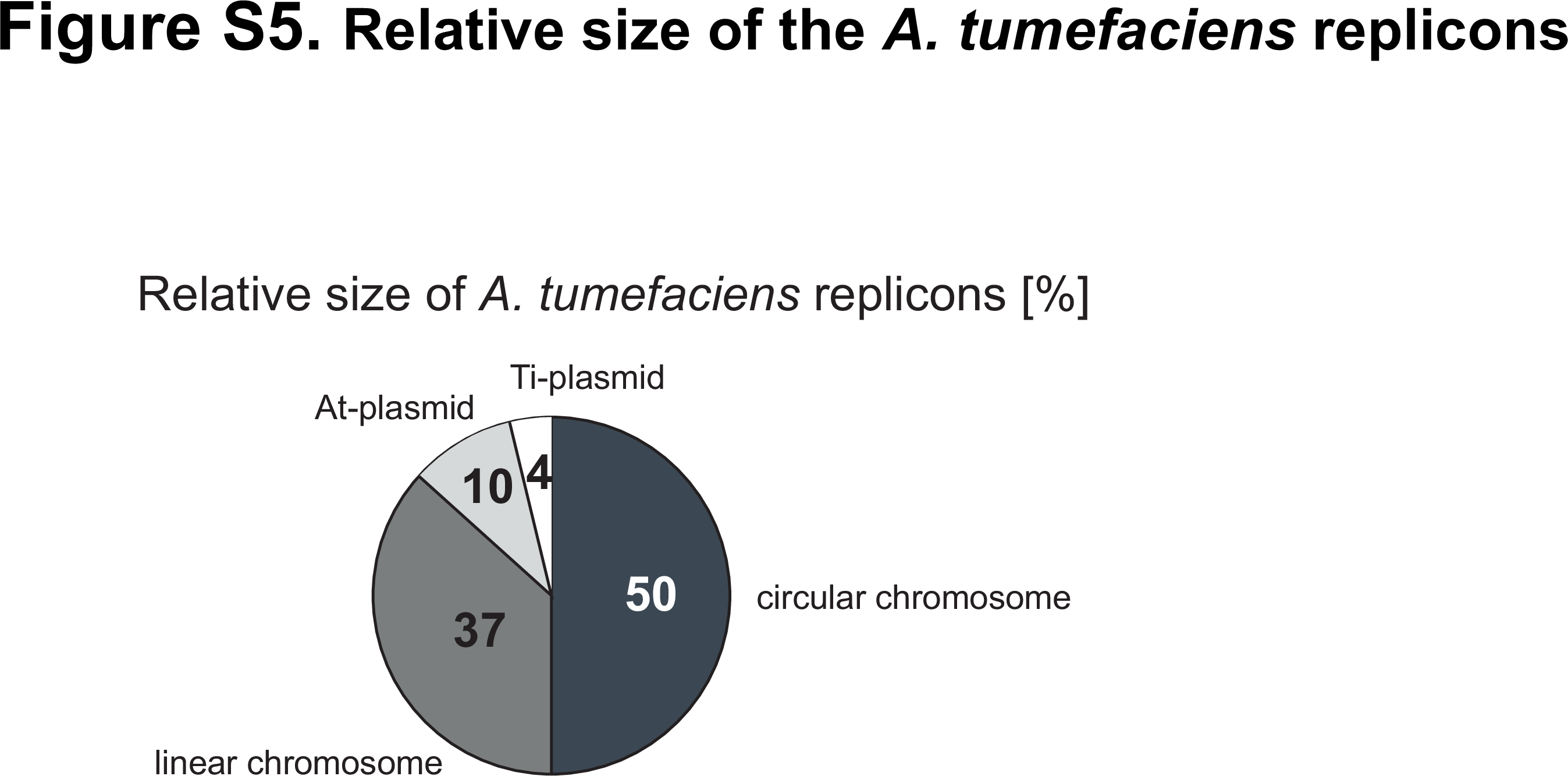

Supplement: Figure S5 — Relative size of the A. tumefaciens replicons. (TIFF) [file pone.0110427.s005.tiff]
